# hgvs: A Python package for manipulating sequence variants using HGVS nomenclature: 2018 Update

**DOI:** 10.1002/humu.23615

**Published:** 2018-09-05

**Authors:** Meng Wang, Keith M. Callenberg, Raymond Dalgleish, Alexandre Fedtsov, Naomi K. Fox, Peter J. Freeman, Kevin B. Jacobs, Piotr Kaleta, Andrew J. McMurry, Andreas Prlić, Veena Rajaraman, Reece K. Hart

**Affiliations:** ^1^ School of Life Sciences Peking University Beijing China; ^2^ University of Pittsburgh Medical Center Pittsburgh Pennsylvania; ^3^ Department of Genetics and Genome Biology University of Leicester Leicester UK; ^4^ Invitae, Inc. San Francisco California; ^5^ Progenity Inc. Ann Arbor Michigan; ^6^ Counsyl South San Francisco California; ^7^ The Apache Software Foundation Los Angeles California

**Keywords:** clinvar, HGVS, sequence variant, variant representation

## Abstract

The Human Genome Variation Society (HGVS) nomenclature guidelines encourage the accurate and standard description of DNA, RNA, and protein sequence variants in public variant databases and the scientific literature. Inconsistent application of the HGVS guidelines can lead to misinterpretation of variants in clinical settings. Reliable software tools are essential to ensure consistent application of the HGVS guidelines when reporting and interpreting variants. We present the hgvs Python package, a comprehensive tool for manipulating sequence variants according to the HGVS nomenclature guidelines. Distinguishing features of the hgvs package include: (1) parsing, formatting, validating, and normalizing variants on genome, transcript, and protein sequences; (2) projecting variants between aligned sequences, including those with gapped alignments; (3) flexible installation using remote or local data (fully local installations eliminate network dependencies); (4) extensive automated tests; and (5) open source development by a community from eight organizations worldwide. This report summarizes recent and significant updates to the hgvs package since its original release in 2014, and presents results of extensive validation using clinical relevant variants from ClinVar and HGMD.

## 1 INTRODUCTION

The standardized representation of genomic, transcript and protein sequence variants is essential in biomedical research and clinical genetics. Accurate interpretation of sequence variants in genetic tests—and, therefore, the resulting patient diagnosis—depends on variants being described, communicated, and compared using consistent representations. The Human Genome Variation Society (HGVS) nomenclature guidelines, first proposed in 1998 (Antonarakis, 1998; den Dunnen & Antonarakis, 2000), have become the de facto international standard for reporting sequence variants (Li et al., 2017; Richards et al., 2015). The guidelines are widely employed in public databases (Fokkema et al., 2011; Landrum et al., 2017), and tools (Cingolani et al., 2012; McLaren et al., 2016; Wang, Li, & Hakonarson, 2010), and they are mandated in most publications and in clinical reports when describing sequence variation.

With the widespread adoption of high‐throughput sequencing and the complexity of DNA, RNA, and protein variants, the HGVS nomenclature has continued to evolve (den Dunnen et al., 2016). Manually generated HGVS representations are prone to applying HGVS nomenclature guidelines incompletely or incorrectly, resulting in malformed representations, incorrect reference bases or incorrect normalization as required by the HGVS nomenclature (Deans, Fairley, den Dunnen, & Clark, 2016; Tack et al., 2016). It is challenging for researchers to manually check all the guidelines in the HGVS nomenclature for each variant discovered in modern sequencing‐based studies. Significant discordance in the reported HGVS representations across four variant annotation tools—ANNOVAR (Wang et al., 2010), Variant Effect Predictor (VEP) (McLaren et al., 2016), SnpEff (Cingolani et al., 2012), and Variation Reporter (https://www.ncbi.nlm.nih.gov/variation/tools/reporter)—demonstrates inconsistencies in the implementations of HGVS (Yen et al., 2017). Thus, automated tools that validate, format, and normalize variants according to the full HGVS nomenclature guidelines are necessary to ensure the accurate communication, comparison, and interpretation of variants. In addition, establishing relationships among variants at the DNA, RNA, and protein level requires the ability to accurately project variants between genomic, transcript (RNA/CDS), and protein sequences.

To facilitate these demands, specialized tools for manipulating HGVS representations of variants according to the HGVS nomenclature guidelines have been developed, including Mutalyzer (Wildeman, van Ophuizen, den Dunnen, & Taschner, 2008) and the hgvs package (Hart et al., 2014). Mutalyzer is a web‐based service for checking the HGVS‐based descriptions of variants and converting variants between genomic level and transcript level. The hgvs package provides a software foundation for parsing, formatting, validating, and mapping DNA, RNA, and protein variants according to the guidelines of HGVS. As with most software, active development is required to address bugs and adapt to evolving needs in the community.

Here, we describe significant updates in the hgvs package since the original release in 2014 and present new results that demonstrate improved performance and functionality of hgvs 1.0. The new hgvs package adds support for inversion, conversion, and identity variants, variant normalization, and multiple assemblies. Variant normalization enables the unique and consistent representation of a variant. The new hgvs release also supports installations with local data sources, resulting in substantial performance gains and eliminating network dependencies. It supports genome assemblies from the NCBI Assembly resource (Kitts et al., 2016), including GRCh37 and GRCh38. The new hgvs package runs on both Python 2.7+ and Python 3.5+. It has been extensively tested with large‐scale and clinically relevant HGVS variant data sets. The upgraded hgvs package provides a flexible, freely available, and easy‐to‐use programming interface for parsing, formatting, validating, mapping, and normalizing of variants described according to HGVS nomenclature.

## 2 METHODS

### 2.1 Package overview

**Figure 1:**
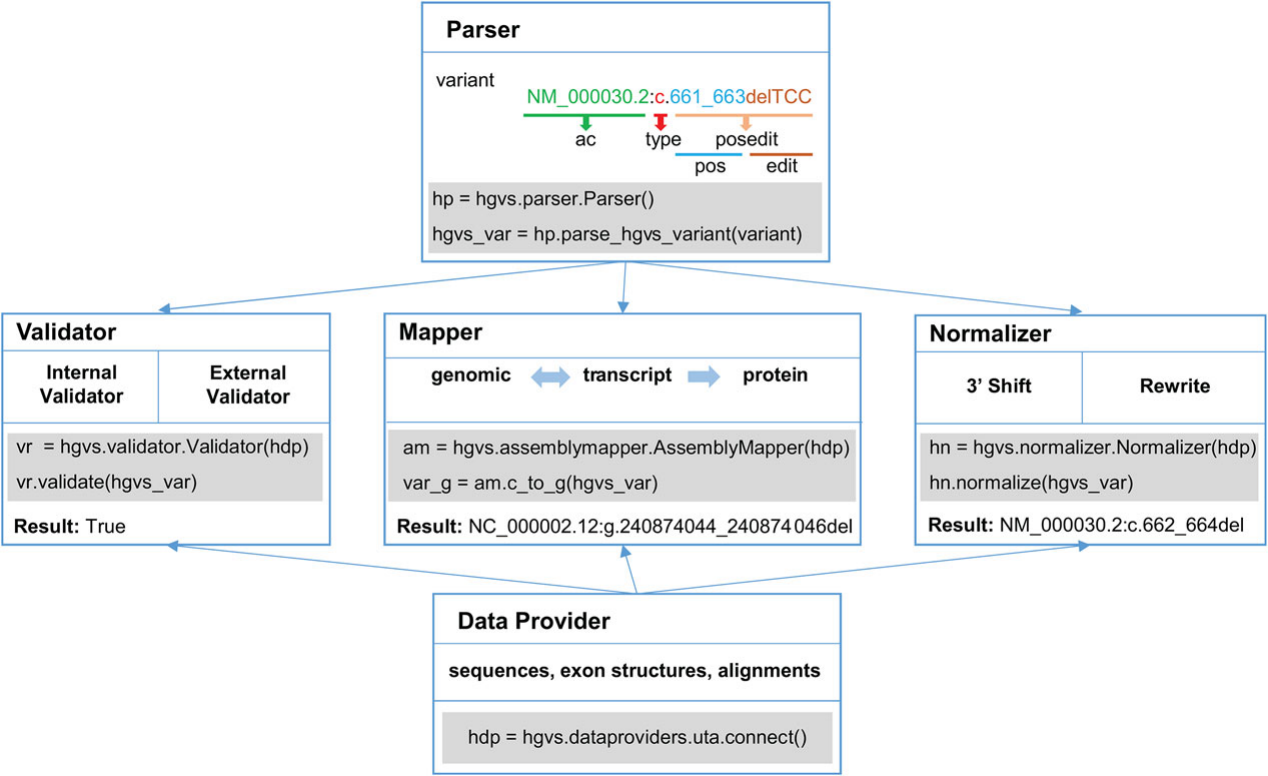
Overview of hgvs package modules and module relationships

The hgvs package is composed of five major modules (Figure 1):

- **parser** module for generating internal object representations of HGVS variant descriptions from string representations,
- **validator** module for checking the validity of variant descriptions,
- **mapper** module for projecting variants between genomic level, transcript level and predicting the effect of variation at the protein level,
- **normalizer** module for shifting and rewriting variants according to the HGVS nomenclature guidelines,
- **data provider** module for querying databases containing reference sequences and annotations such as exon structures required for validating, mapping and normalizing sequence variants.

Changes to these modules are discussed below. The comparisons between the new hgvs package (version 1.2) and the original release (version 0.4) are summarized in Table 1. This release of the hgvs package is consistent with HGVS nomenclature version 15.11, but intentionally omits rarely used aspects of the nomenclature (see Discussion).

**Table 1:** Comparisons between hgvs 1.1 (released in 2018) and hgvs 0.4 (released in 2014)

	v 1.1	v 0.4
parse substitution, delins, insertion, deletion, duplication and repeats	✓	✓
parse inversion, conversion and identity variants	✓	×
strict validation mode	✓	✓
relaxed validation mode	✓	×
different validation response levels	✓	×
supported sequence assemblies	any NCBI Assembly	GRCh37 only
validate variants before mapping	✓	×
normalize (shift and rewrite) variants	✓	×
configurable formatter	✓	×
local data source of transcripts information (UTA)	✓	✓
local data source of sequences (SeqRepo)	✓	×

### 2.2 Key changes since the original release

#### 2.2.1 Parser

The parser in the hgvs package is based on a parsing expression grammar (Hart et al., 2014). In hgvs 1.0, grammar rules were added to support parsing inversion, conversion, and identity variants, in addition to existing support for substitution, deletion–insertion, insertion, deletion, duplication, and repeated sequences. For the unsupported variants types like compound variants, the hgvs package will raise an exception. During parsing, an HGVS‐based variant is parsed into a hierarchical internal object representation (Figure 1). The root representation consists of the reference sequence accession number and version (ac), variant type (type), and the position and change to the reference sequence (PosEdit). The PosEdit object consists of the variant position interval, which contains the starting position and ending position, and the variant change, which is one of the various subclasses of the edit object, which represent the different types of variants. The upgraded hgvs package implements a new BaseOffsetInterval position type to handle CDS positions and to ensure that the start and end position are compatible and comparable, e.g., that the end position is numerically greater than the start position.

#### 2.2.2 Validator

The validator module ensures that a variant is semantically valid and adheres to HGVS guidelines. Validation is performed in two stages: intrinsic validation, which ensures that a variant is internally consistent, and extrinsic validation, which uses external data to verify variant correctness. The intrinsic and extrinsic validators invoke a series of validation criteria, each of which tests a distinct aspect of variant correctness. Intrinsic validation criteria check the validity of information within the variant record, such as requiring that start is less than or equal to end, that the start and end positions are adjacent for an insertion, or that the variant sequence accession is appropriate for the variant type. These criteria are based solely on the variant representation itself and require no additional data. Extrinsic validation criteria use external data to further validate a variant, such as verifying reference sequence agreement and the validity of the variant location. Because extrinsic validation requires external data, it is more computationally expensive and therefore performed after intrinsic validation.

The validation mechanism was significantly refactored in hgvs 1.0. Validators consist of sets of validation criteria that are invoked for a specified variant. Validation criteria now return one of three validation response levels: VALID when all criteria are satisfied, ERROR when a criterion is violated, or WARNING when a criterion cannot be evaluated (discussed below). Validators always raise an exception when any of the validation criteria return ERROR. In addition, validators support a strict mode in which an exception is raised when a criterion returns WARNING.

For example, the extrinsic validator includes a criterion that verifies the agreement of the reference sequence provided in a variant with the sequence implied by the accession and variant location. When these sequences match, the criterion is satisfied (returns VALID). When the sequences do not match, the criterion is violated (returns ERROR). However, an important third case exists: when the variant refers to intronic sequence, which cannot be validated or refuted, the criterion returns WARNING (and an appropriate message). In the default mode, the extrinsic validator would record the WARNING but not raise an exception; in the strict mode, the extrinsic validator would raise an exception. In this way, the hgvs package enables users to distinguish variants that are unambiguously valid, plausibly valid, and unambiguously invalid.

#### 2.2.3 Normalizer

**Figure 2:**
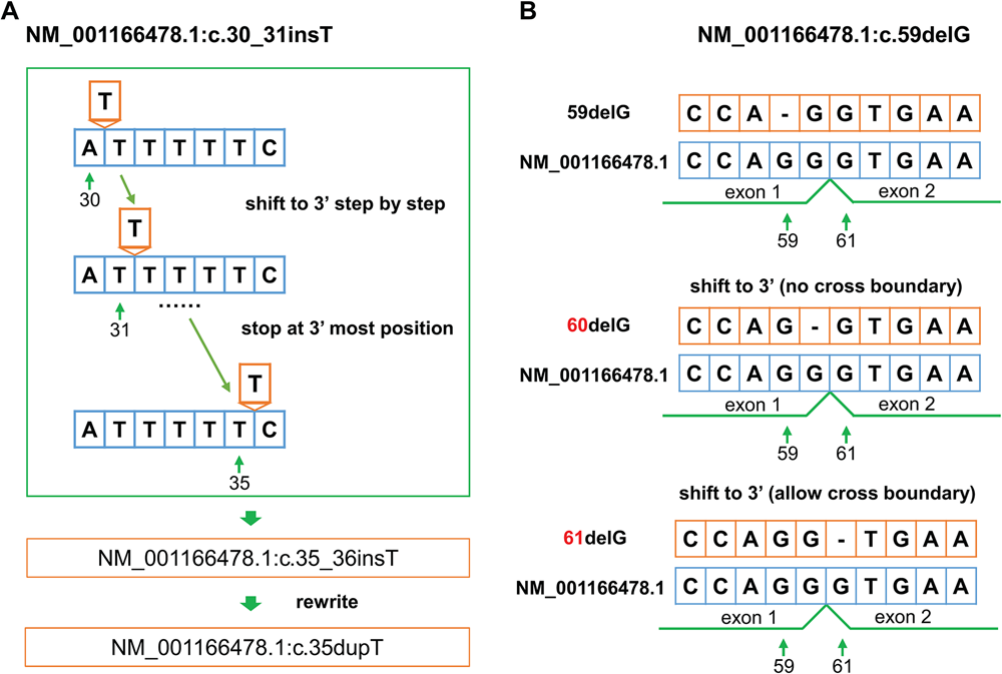
The variant normalization process in hgvs package. a) Normalizing NM_001166478.1:c.30_31insT by 3′ shifting to NM_001166478.1:c.35_36insT, and then rewriting as NM_001166478.1:c.35dupT. b) Normalizing NM_001166478.1:c.59delG by right shifting to or across the intron between c.59 and c.60

A variant may have multiple representations that are syntactically and semantically valid. For example, GCTTTA to GCTTA could be represented as 3delT, 4delT, 5delT, c.3_5delTTTinsTT, c.3_4delTTinsT, but they are semantically the same variants. The normalization process rewrites variants into a canonical form using rules from the HGVS guidelines. The HGVS guidelines require each HGVS‐based variant to be positioned as 3′ as possible and represented by as few nucleotides as possible. In addition, different types of variant descriptions are prioritized. The priority of variant types is substitution > deletion > inversion > duplication > conversion > insertion > deletion–insertion. For instance, when an insertion of A after GCTA is described as 4_5insA, it should be rewritten as 4dupA, according to the HGVS standard, although the two descriptions refer to the same variant. Thus, variant normalization is essential for the standard and consistent description of variants. The upgraded hgvs package implements a normalizer to shift and rewrite variants according to the HGVS nomenclature guidelines (Figure 2).

The hgvs normalizer first trims the common prefix and suffix of reference allele and alternative allele. Next, it utilizes a dynamic extension window starting at the variant position to construct the reference sequence and the altered sequence in the current local window. The normalizer attempts to shift the variant toward the 3′ position in the current window by comparing the reference sequence and the altered sequence (Figure 2A). When the shifted variant reaches the end of current window, the window is extended and the reference sequence and altered sequence in this window are reconstructed. The shifting process continues until the variant is not shiftable or reaches the end of the reference sequence. By default, the hgvs normalizer does not normalize across exon–intron boundaries with respect to transcript reference sequences. The normalizer in hgvs has been extensively tested with variants in border cases, such as variants located at exon‐intron boundaries (Figure 2B).

#### 2.2.4 Formatter

Variant formatting converts an internal object representation into a conventional HGVS textual form. The upgraded hgvs package enables software developers to specify how a variant is formatted (Table 2). Users can specify the maximum reference length to be displayed for deletions. For large deletions that exceed the maximum display sequence length, the reference sequence is omitted from the display. For the formatting of protein variants, it is configurable to use one‐letter or three‐letter (default) representations of amino acids. In addition, stop codons in three‐letter representations may be represented by Ter (default) or ^*^.

**Table 2:** Settings and examples of the configurable formatting in hgvs

Setting	Setting value	Example
**max_ref_length**	0 (default)	NM_001166478.1:c.31_35del
	10	NM_001166478.1:c.31_35delTTTTT
	3	NM_001166478.1:c.31_35del
**p_3_letter**	True (default)	NP_001628.1:p.Gly528Arg
	False	NP_001628.1:p.G528R
**p_term_asterisk**	False (default)	NP_001628.1:p.Gly528Ter
	True	NP_001628.1:p.Gly528^*^

#### 2.2.5 Projection (Mapping)

The variant mapper in hgvs package projects (maps) sequence variants between aligned sequences and predicts the protein level changes with respect to transcript‐level variation. Alignments between transcript and genome sequences often contain sequence discrepancies, including indels, due to sequencing errors in databases and natural sequence variation in populations. A distinguishing feature of the hgvs package is its ability to correctly account for indels between transcript and genome sequences. This ability is critical to accurately interpreting variants in many clinically significant genes (Kalia et al., 2017).

The AssemblyMapper module was added in hgvs 1.0 to significantly streamline projecting variants between genome, transcript, and protein sequences within a single assembly. The module supports any assembly from the NCBI Assembly resource, provided that corresponding genome‐transcript alignment data are available. The AssemblyMapper extends variant mapping abilities in the previous hgvs release by automating (1) the identification of transcripts that span a given genomic variant, (2) the selection of genomic reference sequences for a given transcript variant, (3) the validation before variant projection, and (4) the normalization and reference replacement after variant projection. When projecting a transcript variant to the genome, a transcript may align to two genomic regions, as with pseudoautosomal regions (PARs). The AssemblyMapper permits callers to specify that the X (default) or Y chromosome should be assumed when projecting transcript variants into genomic PARs. Because genome and transcript sequences may contain mismatches, a variant at such a position must be updated to reflect the new reference sequence. Furthermore, the variant might be subject to normalization on the new sequence that was not applicable on the original sequence. For these reasons, the new AssemblyMapper replaces the reference sequence after variant projection, and then normalizes the variant if necessary.

#### 2.2.6 Data provider

**Figure 3:**
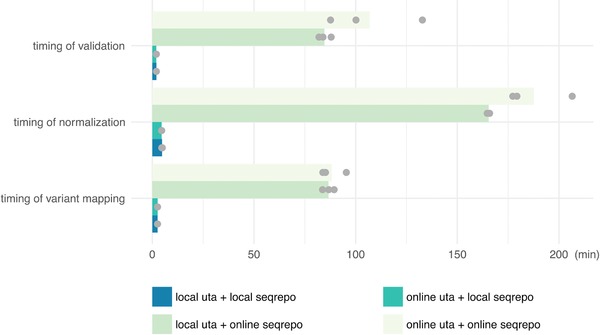
Timing of mapping, normalization and validation of variants with online data sources and local data sources. Each configuration was run three times independently (grey dots). Each bar was the average of all timings

Validating, mapping, and normalizing of variants require reference sequences, exon structures, and transcript‐genome alignments. All network data accesses are provided by a data provider interface. By default, the hgvs package uses a public instance of the Universal Transcript Archive (UTA, https://github.com/biocommons/uta) database to obtain exon structures and transcript‐genome alignments and it retrieves reference sequences from NCBI or EBI via web services. Although these processes are relatively fast (0.1–0.3 s each) and work well for occasional use, the network latency becomes significant for batch processing. The new hgvs package now supports local data sources, thereby eliminating all network access during runtime. By installing the UTA database and sequence repository (SeqRepo, https://github.com/biocommons/biocommons.seqrepo) locally, variant validation, normalization, and mapping are significantly accelerated (Figure 3). Local installation also allows sites to precisely control deployed versions of software and be assured that no patient data are exposed externally. The hgvs privacy statement at https://hgvs.readthedocs.io/en/stable/privacy.html provides details about data that are and are not collected when using public services.

## 3 RESULTS

### 3.1 Effect of local UTA and SeqRepo instances

We evaluated and compared the running time of validation, normalization, and mapping for 100,000 transcript variants in ClinVar (Landrum et al., 2017), using local and remote instances of UTA and local (SeqRepo) and remote sequence sources. The evaluations were run on the same Amazon EC2 m4.xlarge computing instance. Results showed that using local UTA and local SeqRepo could accelerate the validation process 53‐fold, accelerate the normalization process 39‐fold and accelerate the mapping process 34‐fold, compared to that using remote UTA and sequence data sources (Figure 3).

### 3.2 Parsing and validating ClinVar and HGMD variants

To demonstrate the robustness of the upgraded hgvs package, we applied it to batch analyzing transcript variants and genomic variants from ClinVar, which is a trusted large‐scale repository for clinically relevant variants (Landrum et al., 2017). We extracted a total of 284,993 unique transcript variants and 309,899 unique genomic variants from ClinVar release 2017‐05 in XML format. 0.244% Transcript variants and 7.624% genomic variants that have uncertain positions (e.g., NM_000059.3:c.‐227‐?_425+?del) are not currently supported by the hgvs package and were discarded. All the other variants could be parsed by hgvs. We checked the correctness of these parsed variants with hgvs validator. Results revealed that 97.824% of the parsed transcript variants and 99.518% of the parsed genomic variants were valid. The reasons for the invalid variants included syntactic errors, invalid coordinates, discordant deletion/insertion length between positions and edits, and incorrect reference sequences. Table 3 summarizes the results and provides examples of these invalid variants.

**Table 3:** hgvs package parsing and validation results for transcript variants and genomic variants from ClinVar release 2017‐05

	Transcript variants			Genomic variants		
	Number	Percent	Example	Number	Percent	Example
**Variants not considered**						
‐ Variants with uncertain positions	696	0.244%	NM_000059.3:c.‐227‐?_425+?del	23,628	7.624%	NC_000002.12:g.(?_17019)_(774946_?)del
‐ Large variants (>1 M, slow performance)	0	0.000%		58	0.019%	NC_000006.11:g.255350_3189972dup
‐ Sequence data not available	50	0.018%	LRG_219t1:c.3261dupC	40	0.013%	NC_000002.10:g.47852014_47873687del
**Invalid variants**						
‐ Syntactic errors	718	0.252%	NM_000059.3:c.410_411ins8	1,275	0.411%	NC_000017.11:g.43045707delTins6
‐ Invalid coordinates	2,724	0.956%	NM_000038.5:c.^*^2292A>T	0	0.000%	
‐ Discordant del/ins length	4	0.001%	NM_000760.3:c.998_1071del174	6	0.002%	NC_000015.9:g.89382103_89382159del56
‐ Wrong reference	1	0.000%	NM_139058.2:c.333_335dupGCC	0	0.000%	
**Valid variants**				284,892	91.931%	NC_000001.11:g.17028712delTinsCC
‐ Intronic variants	48,797	17.122%	NM_000016.5:c.387+32C>G			
‐ Exonic variants	232,003	81.407%	NM_000059.3:c.201_202dupGA			

Next we applied the hgvs package to analyze the transcript variants with HGVS representations in Human Gene Mutation Database Professional (HGMD pro) (Stenson et al., 2014). The HGMD database is a comprehensive mutation repository for variants related with human inherited diseases. All of the variants in HGMD are manually curated from published literatures. We extracted a total of 165,717 unique transcript variants from HGMD pro release 2016.3. For 10 variants, the transcript data was not available in uta; these variants could not be analyzed. Validation by hgvs revealed that 343 (0.21%) variants had wrong reference bases. The remaining variants (148,056 CDS variants and 17,308 intronic variants) were all valid.

### 3.3 Normalizing ClinVar and HGMD variants

Given the utility of ClinVar and HGMD as resources for clinically relevant variants, standard and uniform representation of variants in ClinVar and HGMD according to the HGVS nomenclature guidelines is critical for the identification and interpretation of disease‐related variants. We first utilized the hgvs normalizer to standardize the representation of transcript variants and genomic variants in ClinVar. Results revealed that 99.622% CDS variants and 95.899% genomic variants were correctly represented according to the HGVS guidelines. 0.306% CDS variants and 4.078% genomic variants did not conform to the 3′ most rule and needed to be shifted to their most 3′ position. 0.092% CDS variants and 0.100% genomic variants did not follow the type priority (substitution > deletion > inversion > duplication > conversion > insertion > deletion–insertion) and could be rewritten, such as rewriting an insertion as a duplication or rewriting a deletion–insertion as an inversion. Table 4 summarizes the normalization results for transcript variants and genomic variants in ClinVar and gives examples for the correctly normalized variant descriptions.

**Table 4:** hgvs package normalization results for transcript variants and genomic variants from ClinVar release 2017‐05

	Transcript variants			Genomic variants		
	Number	Percent	Example	Number	Percent	Example
**Correctly written as‐is**	231,127	99.622%		273,210	95.899%	
**3′ shiftable**	710	0.306%	NM_000041.3:c.291delG ⇒ NM_000041.3:c.292del	11,617	4.078%	NC_000002.12:g.165901915dupA ⇒ NC_000002.12:g.165901920dup
**Rewritable**	213	0.092%	NM_000023.3:c.981_982insGC ⇒ NM_000023.3:c.981_982dup	285	0.100%	NC_000023.11:g.18672015_18672016insA ⇒ NC_000023.11:g.18672015dup
**Both 3′ shiftable and rewritable**	47	0.020%	NM_000183.2:c.3_4insACT ⇒ NM_000183.2:c.5_7dup	220	0.077%	NC_000016.10:g.88865_88866insGT ⇒ NC_000016.10:g.88867_88868dup
**Duplicate variants after normalizing**	30	0.013%	NM_000051.3:c.822delT (RCV000477779) = NM_000051.3:c.824delT (RCV000205636)	55	0.019%	NC_000008.11:g.89955482_89955483delCAinsTG (RCV000486277) = NC_000008.11:g.89955482_89955483invCA (RCV000474157)

Then we employed hgvs to normalize the CDS variants (148,056 in total) in HGMD. The normalization results suggested that 49 variants could be rewritten and one variant should be 3′ shifted (NM_003571.3:c.599_602dupAGGC → NM_003571.3:c.601_604dup). All the other CDS variants (99.97%) were correctly represented.

### 3.4 Round‐Trip projection of ClinVar variants

To test the fidelity of the ability of the hgvs package to project variants between sequence alignments, we undertook “roundtripping” tests in which an original variant was projected from one sequence to another and back; the expectation is that the original and resulting variants should be identical.

In the first test, we projected genomic variants in ClinVar to transcript variants and then back to the original genomic sequence. With the exception of one variant, all the mapped genomic variants were the same as the original genomic variants. The exception is ClinVar variant NC_000003.12:g.46709584_46709610delinsAAGAAGAAGAAGAAGAAGAAGAAGAAG, which is better written as NC_000003.12:g.46709584_46709610 = according to HGVS guidelines. The hgvs package returned the HGVS‐preferred form.

In the second test, we projected transcript variants in ClinVar to genomic sequences and then back to the original transcript. Of 279,568 variants mapped, 42 intronic variants differed from the original transcript variants due to the position offset relevant to the start or end position of the exon. As required by the HGVS guidelines, the hgvs package chooses the closest offset between the end position of directly upstream exon and the start position of the directly downstream exon, while these intronic variants in ClinVar do not conform to this rule. In addition, 29 mapped transcript variants differed from the original transcript variants due to the different exon structures used between hgvs and the reported variants. The remaining 279,497 transcript variants in ClinVar were the same as the cross‐mapping generated transcript variants produced by hgvs.

### 3.5 Important features relative to Mutalyzer

Important correctness differences between hgvs and Mutalyzer (Wildeman et al., 2008) were previously explored (Hart et al., 2014). In this section, we elaborate on the origin of those differences with specific cases. Table 5 summarizes differences between the two packages that affect the accuracy of variant manipulation.

**Table 5:** Important correctness differences between the hgvs package and Mutalyzer. The same input variant was provided to hgvs and Mutalyzer (Mutalyzer 2.0.26, released on July 19, 2017)

Feature	Operation	Input Variant	hgvs result	Mutalyzer result	Explanation
indel‐aware projections	Project transcript variant onto aligned genomic sequence	NM_033089.6:c.460C>N NM_033089.6:c.461C>N	NC_000020.10:g.278687C>N NC_000020.10:g.278691C>N	NC_000020.10:g.278687C>N NC_000020.10:g.278688C>N	NM_033089.6 contains a 3‐nucleotide insertion in the genome relative to the transcript between transcript sequence position 484 and 485 (c.460 and c.461), corresponding to g.278687 and g.278691 on NC_000020.10. Mutalyzer will incorrectly compute coordinates after c.484. This issue affects 428 genes and 1104 transcripts in GRCh37, and 131 genes and 336 transcripts in GRCh38.
validate variants before projection	Project transcript variant onto aligned genomic sequence	NM_003002.3:c.500000G>T	Exception raised (“HGVSError: The given coordinate is outside the bounds of the reference sequence.”)	NC_000011.9:g.112465214G>T	hgvs refuses to extrapolate positions beyond the bounds of the sequence alignment. Mutalyzer does not check sequence bounds.
replace reference sequence after projection	Project transcript variant onto aligned genomic sequence	NM_000024.5:c.46A>T	NC_000005.9:g.148206440G>T	NC_000005.9:g.148206440A>T	NM_000024.5:c.46 corresponds to NC_000005.9:g.148206440, the site of a known SNP (rs1042713). The reference nucleotides in the transcript and genomic sequence are A and G respectively. hgvs replaces the genomic reference sequence after projection, while Mutalyzer does not.
normalize variants after projection	Project transcript variant onto aligned genomic sequence	NM_024426.4:c.1137_1141dup	NC_000011.9:g.32417913_32417917dup	NC_000011.9:g.32417911_32417915dup	NM_024426.4 is on the ‐ strand. The input variant is correctly normalized (3′ shifted). After projection to the genomic sequence, the variant can be normalized on the + strand by 2 nucleotides. Mutalyzer appears to not apply normalization after converting positions. https://groups.google.com/forum/#!topic/hgvs-discuss/M8FUdJ-WCDI.
rewrite variants in preferred forms	Normalize/rewrite variant	NM_001166478.1:c.35_36insT	NM_001166478.1:c.35dup	website warning	hgvs rewrites NM_001166478.1:c.35_36insT as NM_001166478.1:c.35dup. Mutalyzer raises a warning but does not correct the error.

#### 3.5.1 Indel‐aware alignment

Due to polymorphisms and sequencing errors, a small number of transcript‐genome alignments contain substitutions or indels. As of July 2017, 1,104 RefSeq transcripts (428 genes) had gapped alignments with GRCh37 references, and 336 transcripts (131 genes) had gapped alignments with GRCh38 references. The projection algorithm in the hgvs package uses alignment information from NCBI to account for indels within sequence alignments. (Alignment information comes from gff3 files in http://ftp://ftp.ncbi.nlm.nih.gov/refseq/H_sapiens/alignments.) For example, NM_033089.6 contains a three‐nucleotide insertion in the genome relative to the transcript between transcript sequence position 484 and 485 (c.460 and c.461), corresponding to g.278687 and g.278691 on NC_000020.10.

The hgvs package projects the adjacent variants NM_033089.6:c.460C>N and NM_033089.6:c.461C>N to non‐adjacent variants NC_000020.10:g.278687C>N and NC_000020.10:g.278691C>N, consistent with the three‐nucleotide insertion. Mutalyzer projects the same variants to adjacent genomic variants NC_000020.10:g.278687C>N and NC_000020.10:g.278688C>N.

#### 3.5.2 Validating variants before projection

The hgvs package validates variants before projection in order to ensure that algorithms are applied in appropriate contexts. For example, it refuses to project variants with invalid coordinates. When projecting NM_003002.3:c.500000G>T to genomic sequence, hgvs will raise an error signaling that the nucleotide coordinate is out of bounds. However, Mutalyzer will project this variant to NC_000011.9:g.112465214G>T, nearly 500 MB from the transcript.

Projecting variants in the vicinity of sequence substitutions and indels is fraught with many challenges. The variant mapper in the new hgvs package is designed to deal with possible cases when projecting variants located at transcript‐genome alignment gaps and substitutions. Extensive tests demonstrate the new hgvs package could correctly project such variants between transcript and genome sequences. Table 6 summarizes hgvs results of projecting variants that are within, exactly cover, partially cover and extend beyond the bounds of the transcript‐genome alignment gaps.

**Table 6:** Projection of variants in the vicinity of transcript–genome alignment gaps

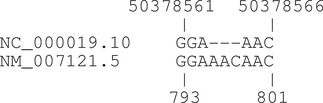		
Position relative to gap	Variant	Transcript to genome projection
**within**	NM_007121.5:n.796A>T	NC_000019.10:g.50378563_50378564insTAC
	NM_007121.5:n.796_797del	NC_000019.10:g.50378563_50378564insC
	NM_007121.5:n.796_797insT	NC_000019.10:g.50378564_50378565insTACA
**exact**	NM_007121.5:n.796_798del	NC_000019.10:g.50378 565_50378567dup
	NM_007121.5:n.796_798delinsTC GG	NC_000019.10:g.50378563_50378564insTCGG
**partial**	NM_007121.5:n.795_796del	NC_000019.10:g.50378563_50378564insC
	NM_007121.5:n.795_796delinsTT	NC_000019.10:g.50378563delinsTTAC
	NM_007121.5:n.795_796insT	NC_000019.10:g.50378563_50378564insTAAC
**span**	NM_007121.5:n.794_800del	NC_000019.10:g.50378562_50378565del
	NM_007121.5:n.794_800delinsTC	NC_000019.10:g.50378562_50378565delinsTC

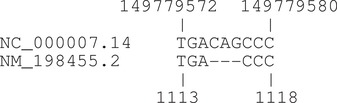		
Position relative to gap	Variant	Transcript to genome projection
**within**	NM_198455.2:n.1115_1116insT	NC_000007.14:g.149779575_149779577delinsT
**exact**	NM_198455.2:n.1115_1116insCAG	
	N M_198455.2:n.1115_1116=	NC_0 00007.14:g.149779576_149779578del
**partial**	NM_198455.2:n.1115_1116insCA	NC_000007.14:g.149779577del
**span**	NM_198455.2:n.1114_1117del	NC_000007.14:g.149779573_149779579del
	NM_198455.2:n.1114_1117delinsCA	NC_000007.14:g.149779573_149779579delinsCA

#### 3.5.3 Updating variant reference sequence after projection

When there are substitution differences between transcript and genomic sequence, the variant reference sequence must be updated to reflect the correct sequence. For example, NM_000024.5:c.46 corresponds to NC_000005.9:g.148206440, the site of a known SNP (rs1042713). The reference nucleotides in the transcript and genomic sequence are A and G, respectively. When hgvs projects NM_000024.5:c.46A>T to NC_000005.9:g.148206440G>T, it has replaced the reference A with G. Mutalyzer returns NC_000005.9:g.148206440A>T, which is invalid.

#### 3.5.4 Normalizing variants after projection

When projecting variants between sequences, it is necessary in some circumstances to renormalize the variant, especially for transcripts on the minus strand because 3′ normalization will operate in the opposite direction to the genomic sequence (plus strand). For example, consider NM_024426.4:c.1137_1141dup. On NM_024426.4, the variant is correctly normalized in the 3′ direction. Before normalization, the variant projects to NC_000011.9:g.32417911_32417915dup, the result returned by Mutalyzer. This variant is not properly normalized (3′ shifted). The dup sequence (on the plus strand) is ACCGT and the trailing sequence is ACAAG; the dup must be right shifted by the common prefix (AC), to result in NC_000011.9:g.32417913_32417917dup, which is the result returned by hgvs. To be clear, inserting ACCGT after 32417915 (NC_000011.9:g.32417911_32417915dup) has the same net effect as inserting CGTAC after 32417917 (NC_000011.9:g.32417913_32417917dup).

#### 3.5.5 Rewriting variants in preferred forms

In addition to 3′ shifting, the normalizer algorithm in hgvs rewrites variants according to this priority scheme: substitution > deletion > inversion > duplication > conversion > insertion > deletion‐insertion. For example, a deletion‐insertion variant should be written as an insertion if possible. Consider the variant NM_001166478.1:c.35_36insT. Since c.35 is a T, this variant is better written as NM_001166478.1:c.35dup. The hgvs package will generate the dup form upon normalization, whereas Mutalyzer will raise an error (but not rewrite the variant).

#### 3.5.6 Additional differences

A broader comparison of features of hgvs and Mutalyzer is summarized in Supporting Information Table S1.

## 4 DISCUSSION

We have presented the upgraded hgvs package, a comprehensive tool to manipulate variant representations according to the HGVS nomenclature. The new hgvs package supports most variant types and a comprehensive set of manipulations of those variants. We demonstrated a high concordance of projection and normalization functions using 284,993 unique transcript variants and 309,899 unique genomic variants from ClinVar; where these were discordant, the hgvs package generated the representation preferred by HGVS guidelines. We have also reported on difficult cases where the hgvs package generates a correct variant representation whereas Mutalyzer does not. The new hgvs package supports the use of local data sources, which accelerates validation, projection, and normalization operations by 30–50‐fold relative to the previous version that relied on remote sequence data.

The hgvs package is known to have been used in at least three distinct applications relevant to next generation sequencing (NGS) analysis pipelines. First, the hgvs package provides a reliable mechanism to project published transcript variants to genomic coordinates. These genomic coordinates may be used to identify regions for targeted assays. Second, variants discovered in genomic sequence may be projected onto transcript sequences and, for coding transcript sequences, also onto protein sequences. Modern pipelines supporting NGS‐based clinical genetics usually start with an effect predictor like ANNOVAR (Wang et al., 2010), VEP (McLaren et al., 2016), or SnpEff (Cingolani et al., 2012). However, significant discordances exist in the reported HGVS representations across these tools (Yen et al., 2017). The hgvs tool could be employed following these annotation tools to generate unique and standardized variant representations according to the HGVS nomenclature. Third, the hgvs package provides a mechanism to generate a family of variants related to a seed variant—colloquially, all other written forms of a given variant—that may be subsequently used as queries for supporting evidence during interpretation or variant curation.

The hgvs package is an open‐source project driven by the community, with contributions from 13 developers at eight different organizations worldwide. The code is extensively tested with unit tests covering all functions, and functional tests with manually composed extreme examples and variants in complex sequence contexts (code coverage is 92%). Every commit to the code triggers automatic testing to ensure the robustness of the tool. All bugs and feature requests are available publicly (https://github.com/biocommons/hgvs/issues). Importantly, many of the features in this release were requested by users. A public mailing list (https://groups.google.com/forum/#!forum/hgvs-discuss) and real‐time chat (https://gitter.im/biocommons/hgvs) enable users and developers to discuss issues.

The hgvs package is easy to install (via Python's pip command). Fully local installations are straightforward and obviate all network access. The upgraded hgvs package is developed and tested on Python 2.7+ and Python 3.5+. The Python API provides a flexible software foundation on which to build data processing pipelines or graphical interfaces, such as VariantValidator (Freeman, Hart, Gretton, Brookes, & Dalgleish, 2018; https://variantvalidator.org/).

The hgvs project evolves as the HGVS guidelines evolve. The hgvs package does not currently support compound, mosaic, chimeric, or translocation variants, and it does not support ISCN extensions. The project roadmap, which is shared publicly at github, includes adding support for variants with uncertain CDS offsets and support for compound, mosaic, and chimeric variants.

The hgvs package is a robust tool for working with HGVS variants. By making hgvs freely available for commercial and noncommercial uses, and by providing support for fully local installations, we have provided a flexible, clinical‐grade toolkit that contributes to the accurate interpretation of variants for patients and the consistent description of HGVS variants in public databases.

## 5 AVAILABILITY

The hgvs package is available at github (https://github.com/biocommons/hgvs) under the Apache 2.0 open‐source license. Python packages are available at PyPI (https://pypi.python.org/pypi/hgvs) and easily installed ‘pip install hgvs’. Docker images are available at docker hub (https://hub.docker.com/r/biocommons/hgvs/). Extensive documentation is available (https://hgvs.readthedocs.io/en/master/index.html).

## Supporting information

Supporting information

Supporting information
