# Supplementary material for: hgvs: A Python package for manipulating sequence variants using HGVS nomenclature: 2018 Update
Source: Hum Mutat. 2018 Sep 5;39(12):1803–13. doi: 10.1002/humu.23615 (PMC6282708; doi:10.1002/humu.23615)
Supplement: Supplementary file 2 — Supporting information [file HUMU-39-1803-s002.docx]

# **Supplemental Data**

**Table S1.** Feature comparison of hgvs package and mutalyzer.

| **feature** | **hgvs** | **mutalyzer** | **comment** |
| --- | --- | --- | --- |
| Core Capabilities |  |  |  |
| variant parsing | ✓ | ✓ | hgvs parses variants into a typed object model, whereas Mutalyzer parses into a set of attributes ("RawVar"). The hgvs package does not support chimeric variants or mosaic variants. |
| variant validation | ✓ | ✓ | Mutalyzer name checker does not validate genomic sequences (the website cannot read reference files more than 10 MB in size) |
| variant projection/position conversion | ✓ | ✓ | hgvs uses an indel-aware mapper. Mutalyzer returns incorrect results when the transcript-genome alignment contains gaps. |
| variant normalization | ✓ | × | Mutalyzer will "roll forward" variants, but does not provide normalization as a distinct operation. For example, hgvs shifts NM_001166478.1:c.31del as NM_001166478.1:c.35del. Mutalyzer raises a warning but does not correct the error. |
|  |  |  |  |
| Interfaces |  |  |  |
| Web site | ✓ | ✓ | VariantValidator, a distinct project, provides a sophisticated web interface to hgvs functionality. Mutalyzer is a web interface with integrated variant functionality. |
| Programmatic interface | API | SOAP | The hgvs API is available through a Python library, but does not provide a web service (e.g., REST) interface. Conversely, Mutalyzer provides a SOAP web service interface, but is not available as a library. |
|  |  |  |  |
| Important differences |  |  |  |
| indel-aware projections | ✓ | × | hgvs uses an indel-aware mapper. Mutalyzer returns incorrect results when the transcript-genome alignment contains gaps. For example, for NM_033089.6:c.571C>G, hgvs correctly returns NC_000020.10:g.278801C>G whereas Mutalyzer returns NC_000020.10:g.278798C>G due to a 3-nucleotide insertion in the transcript (relative to the genome). |
| normalize variants during projection | ✓ | × | hgvs projects NM_001166478.1:c.33_34insT to NC_000006.11:g.49917127dup. Mutalyzer projects it to NC_000006.11:g.49917124_49917125insA (unshifted and not rewritten). Also see https://groups.google.com/forum/#!topic/hgvs-discuss/M8FUdJ-WCDI. |
| validate variants during projection | ✓ | × | hgvs will not project NM_003002.3:c.27400G>T to genomic sequence because the position is beyond the bounds of the transcript. Mutalyzer will project it to genomic sequence. |
| variant rewriting | ✓ | × | hgvs rewrites NM_001166478.1:c.35_36insT as NM_001166478.1:c.35dup. Mutalyzer raises a warning but does not correct the error. |
| reference replacement | ✓ | × | When projecting NM_000024.5:c.46A>T to chromosome 5, hgvs returns NC_000005.9:g.148206440G>T (G is correct genomic reference), whereas Mutalyzer returns the NC_000005.9:g.148206440A>T (invalid reference nucleotide). |
|  |  |  |  |
| Software development |  |  |  |
| Python version compatibility | 2.7+,  3.5+,  3.6+, | 2.7+ |  |
| Python package available in PyPi | ✓ | × | Packages in pypi may be installed simply (pip install hgvs). Installing mutalyzer requires fetching source code. |
| Source code | github | github | https://github.com/biocommons/hgvs  https://github.com/mutalyzer/mutalyzer |
| Testing coverage | 92% | ? | hgvs code coverage is 92% and publicly visible. |
| Continuous integration and deployment | ✓ | × | Every commit in hgvs is tested; on success, packages are deployed to pypi. |
| isolated installation available | ✓ | ✓ |  |
| commits since Jan 1, 2017 | 198 | 32 |  |
| contributors since Jan 1, 2017 | 7 | 4 |  |
| license | Apache | Affero | The Apache license permits uses by any person or organization without encumbrances to derivative or combined works. The Affero license requires release of any modifications. |

**Supp. Data S2. Instructions for results database**

The SQLite database contains results from normalizing variants in ClinVar 2017-05.

The MD5 checksum of the database is shown in the example.

Example:

| snafu$ md5sum clinvar.sqlite  11c4586c8286e981b99d7460908186cb clinvar.sqlite  snafu$ sqlite3 clinvar.sqlite  -- Loading resources from /home/reece/.sqliterc  SQLite version 3.19.3 2017-06-08 14:26:16  Enter ".help" for usage hints.  sqlite> select count(var_c) from clinvar where var_c is not null;  count(var_c)  ------------  284993 |
| --- |
